# A pilot randomized controlled trial comparing noradrenaline and adrenaline as a first-line vasopressor for fluid-refractory septic shock in neonates

**DOI:** 10.3389/fped.2024.1443990

**Published:** 2024-10-03

**Authors:** Reema Garegrat, Suprabha Patnaik, Sonali Suryawanshi, Chinmay Chetan, Nishant Banait, Pari Singh, Aditya Kallimath, Naharmal B. Soni, Yogen Singh, Pradeep Suryawanshi

**Affiliations:** ^1^ Department of Neonatology, Bharati Vidyapeeth Hospital and Research Institute, Pune, India; ^2^ Department of Pharmacology, Bharati Vidyapeeth Hospital and Research Institute, Pune, India; ^3^ Department of Neonatology, Himalayan Institute of Medical Sciences, Swami Rama Himalayan University, Dehradun, India; ^4^ Department of Neonatology, AIIMS, Nagpur, India; ^5^ Department of Neonatology, Sidra Medicine, Doha, Qatar; ^6^ Department of Pediatrics, Division of Neonatology, University of California Davis Children's Hospital, Sacramento, CA, United States

**Keywords:** adrenaline, neonate, noradrenaline, sepsis, septic shock

## Abstract

**Background and study design:**

Limited data exists on noradrenaline therapy in neonatal septic shock. We compared the efficacy of noradrenaline with adrenaline in neonatal septic shock. This single center, open label, pilot randomized controlled trial included neonates with clinical evidence of sepsis and shock.

**Study outcomes:**

Primary outcomes were: 1) resolution of shock one hour after treatment, and 2) mortality during hospital stay. Secondary outcomes included: need for additional vasopressors; hemodynamic stability without further administration of vasopressors for ≥2 h; changes in blood pressure and heart rate after 1 h of vasopressor treatment; and morbidities during the hospital stay.

**Results:**

Of 65 eligible neonates, 42 were randomized (21 each in adrenaline and noradrenaline treatment arms) between August 2020 and January 2022, at level III neonatal intensive care unit (NICU) of Bharati Vidyapeeth Deemed University Medical College and Hospital (BVDUMCH). The mean (SD) gestational age and mean (SD) birth weight were 36.1(4.2) weeks and 1.8 (0.2) kilograms birth weight for noradrenaline and 36.9 (4.1) weeks and 1.7 (0.7) kilograms for adrenaline. Shock resolved within 1 h of vasopressor therapy in 76.2% neonates in the noradrenaline arm and 61.9% in adrenaline arm (*p* value-0.53). Mortality during hospital stay was 28.6% (6/21) in noradrenaline group and 33.3% (7/21) in adrenaline group (*p* value- 0.58). Additional vasopressors were required in 23.8% neonates of the noradrenaline group compared to 38.1% neonates in adrenaline arm (*p* value-0.53). Median (SD) duration of intensive care stay was 6 (SD) days in the noradrenaline group and 10 (SD) days in the adrenaline group (*p* value-0.045).

**Conclusion:**

Among neonates with septic shock, the efficacy of noradrenaline was comparable to adrenaline in resolving septic shock after one hour of infusion and on the mortality during hospital stay.

**Clinical Trial Registration:**

https://ctri.nic.in/Clinicaltrials/pmaindet2.php?EncHid=NDI2NTc=&Enc=&userName=noradrenaline, Clinical Trials Registry – India with identifier CTRI/2020/08/027185 (17/08/2020).

## Introduction

Sepsis remains a leading cause of neonatal morbidity and mortality (1). Fluid-refractory septic shock, an important consequence of sepsis, is managed according to guidelines developed for intensivists and pediatricians. The surviving sepsis campaign international guidelines for the management of septic shock and sepsis-associated organ dysfunction in children of the society of critical care medicine does not recommend any specific drug as the first-line vasopressor agent and instead suggests either adrenaline or noradrenaline can be used and guided by “clinician preference, individual patient physiology, and local system factors” (2). The American College of Critical Care Medicine/Pediatric Advanced Life Support Clinical Guidelines for hemodynamic support of neonates and children with septic shock recommends adrenaline as the first-line agent to begin with but shifting to noradrenaline in case of vasodilatory shock (3). Neonatal sepsis studies have shown mild cardiac dysfunction in infants with neonatal septic shock and some developing pulmonary hypertension (4, 5). The newborns present with a haemodynamic pattern similar to children and adults with warm shock or cold shock, depending upon the compensatory mechanisms. In such a clinical scenario, choice of cardiovascular medication is not clearly defined. In neonatal septic shock, although adrenaline is commonly used but noradrenaline can give additional benefit of pulmonary pressure reduction, decrease in peripheral vasodilatation and thus increasing blood pressure. However, noradrenaline is often not used as first line therapy based on the assumption that use of noradrenaline may further compromise left ventricular function in infants with moderate to severe cardiac dysfunction. There are no guidelines as of now for the usage of the drugs interchangeably. Considering this, the current study was designed to compare noradrenaline and adrenaline as the first-line vasoactive medication in neonatal septic shock to objectively see the effectiveness and the hemodynamic impact from use of either of these drugs.

## Material and methods

### Study ethics and registration

The study was approved by the institutional review board of Bharati Vidyapeeth Deemed University Medical College and Hospital (BVDUMCH), Pune, India, and registered in Clinical Trials Registry – India with identifier CTRI/2020/08/027185 dated 17/08/2020. Informed consent was obtained from parents of the neonates for their enrolment in the study.

### Study design

The open label, parallel arms, randomized study was conducted in the level III neonatal intensive care unit (NICU) of BVDUMCH between August 2020 and January 2022.

### Sample size calculation

Successful resolution of shock within one hour of adrenaline occurred in around 25% of the neonates (6). To detect an increase in resolution from 25% in the adrenaline group to 60% in the noradrenaline group, at 80% power and 0.05 significance, 30 neonates per arm (60 total) were to be recruited. However, the trial was discontinued after recruitment of 42 neonates due to the COVID pandemic.

### Study population

Neonates admitted in the NICU with fluid-refractory septic shock were eligible for enrollment. Septic shock was defined as systemic hypotension with decreased perfusion: mean blood pressure < 10th percentile of the normal for birth weight (BW) and postnatal age for Indian children (7) with at least three of these five criteria satisfied: resting heart rate > 20 above normal, feeble or non-palpable peripheral pulses, pale or bluish discoloration of palmar/plantar surfaces, palmar/plantar capillary refill time > 3 s, and urine output < 1 ml/kg/h. Neonates with congenital malformations, hypovolemic shock, cardiogenic shock, clinically suspected or echocardiographically proven congenital heart disease including hemodynamically significant patent ductus arteriosus (hsPDA), moderate to severe hypoxic ischemic encephalopathy with blood pH < 7 at birth or APGAR score < 5 at 5 min, or those already on inotropic or vasopressor drugs were excluded. HsPDA was defined as duct diameter >1.6 mm with retrograde descending aorta flow with Qp/Qs > 1.5.

Over the study period of 22 months, 42 of the 65 neonates considered eligible for the study were enrolled after parental consent. Twenty neonates receiving other inotropes and three with congenital anomalies were excluded from the study.

### Intervention and clinical care

At baseline, demographic and clinical data, including hemodynamic status (extremity perfusion, mental status, heart rates, and BP) was recorded in a pre-designed form. The first dose of broad-spectrum antibiotics was administered within 1 h of sepsis recognition, and samples were collected for relevant cultures, blood gas analyses, and lactate measurements.

Forty two enrolled neonates were randomized using computer generated random numbers (Random Allocation Software) (8) to receive either adrenaline or noradrenaline (21 in each group). The neonates first received a fluid bolus of 10 ml/kg of crystalloids, after which functional echocardiography was performed. Continuous infusion of adrenaline or noradrenaline was then started at 0.1 or 0.2 μg/kg/min, respectively, via peripheral venous access. If there was no hemodynamic response to the initial dose, vasopressor dose was incremented to 0.3 or 0.5 μg/kg/min, respectively by increasing the fluid flow rate up to two times during 20 min. Reduction of adrenaline/noradrenaline in steps of 0.1 μg/kg/min was initiated as per the treating neonatologist after resolution of shock. In the case of no response to the highest possible study drug dose, the selection of another vasopressor was left to the physician's discretion. Functional echocardiography and arterial lactate assay were performed at baseline and at 1 h after start of treatment with the study drugs. Urine output was measured hourly till the withdrawal of vasoactive drugs. Data on fluid balance, blood and body fluid cultures, serologic assays, respiratory support, and antibiotic therapy were recorded.

Serious adverse events of tachycardia, hypertension, arrhythmia, renal ischemia, pulmonary oedema, hyperglycaemia, and hypokalaemia during infusion of the study drugs were recorded. Blood pressure was monitored by a non-invasive oscillometric method (Nihon Kohden VISMO multipara monitor). Echocardiography was performed by a single investigator using Phillips 50G with 12-4 MHz high-frequency phased array transducer probe using published methods at the beginning after fluid bolus and again 60 min later. Functional parameters were measured twice in the echocardiography procedures.

### Outcome measures

Primary outcomes were: (1) resolution of shock one hour after treatment, and (2) mortality during hospitilisation. Secondary outcomes included: need of additional vasopressors; hemodynamic stability without further administration of vasopressors for ≥2 h; changes in blood pressure and heart rate after 1 h of vasopressor treatment; and morbidities during the hospital stay in terms of duration of ventilation support and incidence of acute kidney injury (AKI), cerebellar or intraventricular hemorrhage (IVH), nosocomial infection, periventricular leukomalacia (PVL), and stage II/III necrotizing enterocolitis (NEC).

### Statistical analysis

Data was analysed using SPSS software (version 21). Standard t test was used for two-group comparisons of continuous variables. Chi square test was used for categorical variables, unless observed frequency in at least one cell was <5, in which case Fisher's exact test was used.

## Results

A total of 2,035 neonates were admitted in the NICU during the study period (August 2020 to January 2022). Of these, 197 neonates (9.7%) had sepsis and 65 (33.0%) of them developed septic shock. There were 4 culture positive neonates in the noradrenaline arm and 8 culture positive cases in the adrenaline arm. 4 organisms in the noradrenaline group were in the blood, namely klebsiella pneumoniae, pseudomonas aueruginosa, eneterobacter cloacae and acinetobacter baumanni. The 8 organisms in the adrenaline group were in the blood, namely 2 klebsiella pneumoniae, 1 escherichia Coli, 1 acineobacter baumanii, 2 strenotropomonas maltophilia, 1 enterobacter cloacae and 1 serratia marcescens.

**Figure 1:**
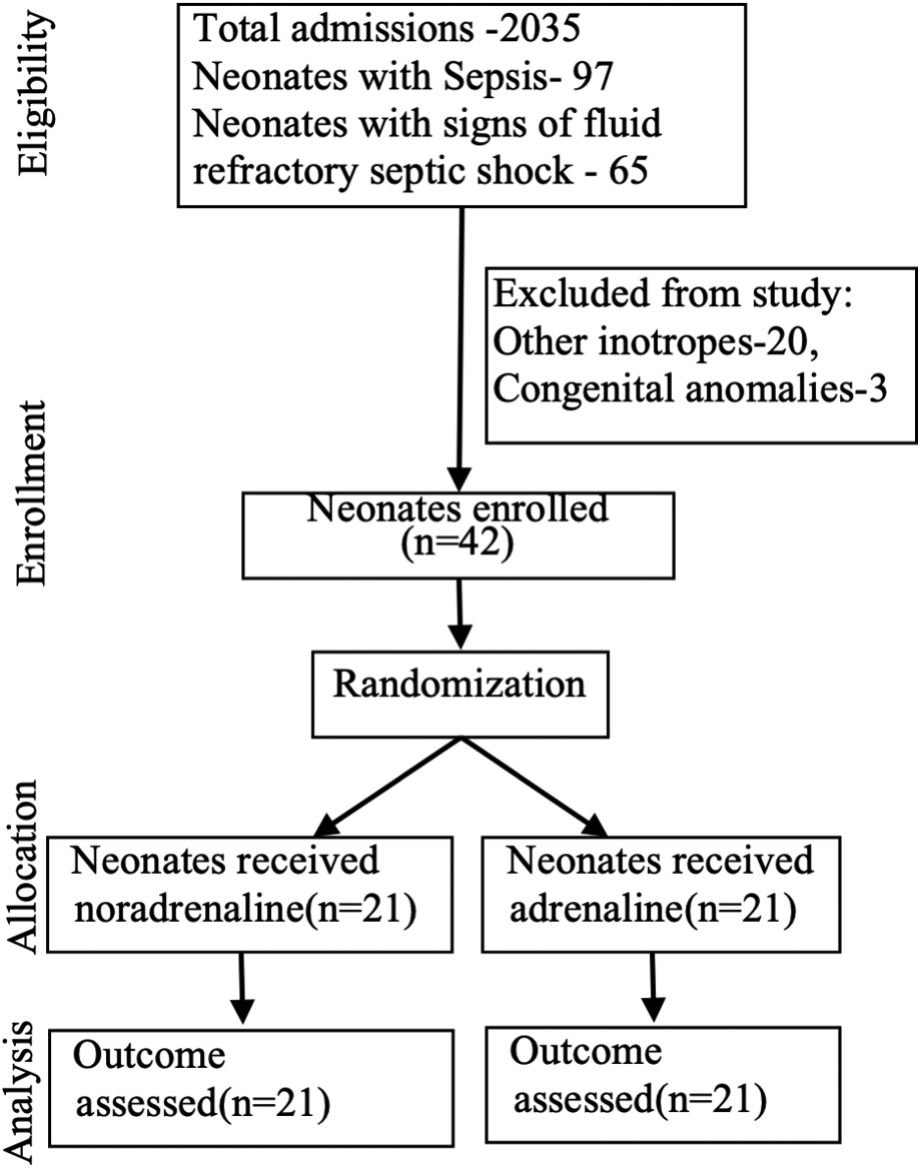
Study flowchart.

Forty-two of the 65 neonates eligible were infants with signs and symptoms suggestive of septic shock that got enrolled in the study and were randomized into two groups: 21 cases in each group receiving either adrenaline or noradrenaline as the first-line vasopressor treatment (Figure 1). The two groups were similar at baseline in terms of important maternal and neonatal characteristics (Table 1), clinical features (Table 1), and echocardiographic findings of ventricular function (Table 2A).

**Table 1:** Characteristics of the study groups.

	Adrenaline (*n* = 21)	Noradrenaline (*n* = 21)	*p*-value^a^
Maternal characteristics			
Primigravida, *n* (%)	10 (47.6%)	12 (57.1%)	0.64
Anemia, *n* (%)	3 (14.3%)	3 (14.3%)	1.00
Pregnancy-induced hypertension, *n* (%)	2 (9.5%)	2 (9.5%)	1.00
Cesarian delivery, *n* (%)	17 (81.0%)	13 (61.9%)	0.31
Antenatal steroids, *n* (%)	16 (76.2%)	15 (71.4%)	1.00
Neonatal characteristics			
Male sex, *n* (%)	13 (61.9%)	17 (81.0%)	0.31
Gestational age (w), mean (SD)	36.9 (4.1)	36.1 (4.2)	0.21
Birth weight (kg), mean (SD)	1.9 (0.7)	1.8 (0.2)	0.64
APGAR at 5 min, mean (SD)	7.8 (1.8)	7.3 (1.8)	0.49
Clinical features			
Hypotension, *n* (%)	21 (100%)	20 (95.2%)	1.00
Cold extremities, *n* (%)	19 (90.5%)	17 (81.0%)	0.66
Icterus, *n* (%)	17 (81.0%)	17 (81.0%)	1.00
Pallor, *n* (%)	18 (85.7%)	13 (61.9%)	0.08
Weak or non-palpable pulse, *n* (%)	12 (57.1%)	13 (61.9%)	0.76
Low blood O_2_ saturation, *n* (%)	13 (61.9%)	10 (47.6%)	0.53
Edema, *n* (%)	13 (61.9%)	9 (42.9%)	0.22
Tachypnea, *n* (%)	10 (47.6%)	11 (52.4%)	0.76
Seizures, *n* (%)	5 (23.8%)	3 (14.3%)	1.00
Lethargy, *n* (%)	4 (19.0%)	3 (14.3%)	1.00
Bradypnea, *n* (%)	4 (19.0%)	3 (14.3%)	1.00
Refusal to feed, *n* (%)	3 (14.3%)	3 (14.3%)	1.00
Moderate hypothermia, *n* (%)	4 (19.0%)	1 (4.8%)	0.34
Fever, *n* (%)	0 (0%)	1 (4.8%)	1.00
Resuscitation required, *n* (%)	5 (23.8%)	10 (47.6%)	0.22
Duration of shock in hours, median (IQR)	6 (2–8)	4 (2–8)	0.47

^a^ for continuous variables, standard *t* test, and for categorical variables, chi square test or Fisher's exact test in case of any observed frequency ≤ 5.

**Table 2A:** Echocardiographic findings of the study groups at baseline.

	Adrenaline (*n* = 21)	Noradrenaline (*n* = 21)	*p*-value
Left ventricle – mean (SD)			
Heart rate (per min)	153.3 (16.8)	148.6 (16.6)	0.41
Velocity time integral (m)	9.8 (3.9)	9.4 (2.6)	0.74
Aortic Outflow Cross Sectional area (AoCSA) (cm^2^)	6.1 (1.3)	6.0 (1.2)	0.81
Output (ml/kg/min)	212.9 (96.8)	208.8 (83.5)	0.89
Fractional shortening (%)	34.1 (19)	29.0 (13.5)	0.46
Right ventricle – mean (SD)			
Heart rate (per min)	154.0 (17.0)	147.2 (16.5)	0.24
Velocity time integral (m)	10.6 (4.3)	9.9 (3.0)	0.60
Pulmonary Outflow Cross Sectional area (PoCSA) (cm^2^)	6.5 (1.2)	6.3 (1.2)	0.63
Output (ml/kg/min)	268.3 (154.0)	237.0 (134.1)	0.52
Tricuspid annular plane systolic excursion (TAPSE) (mm)	7.5 (3.2)	9.0 (2.6)	0.18

Resolution of shock without a second vasopressor or inotrope was achieved in 76.2% of cases in the noradrenaline group and 61.9% in the adrenaline group (*p*-value = 0.53). Dose increment was needed in 42.9% of cases receiving noradrenaline and 28.6% for adrenaline (*p*-value = 0.33). Mortality during hospitalisation was similar in two groups, 28.6% and 33.3% for noradrenaline and adrenaline, respectively (*p*-value = 1.00). Changes in ventricular function, vital signs, and blood acid-base parameters after one hour of starting the study drug infusion were also similar for the two groups (Table 2B). Mean requirement of fluid boluses was not significantly different for the two groups (1.67 vs. 1.48; *p*-value = 0.46).

**Table 2B:** Echocardiographic findings of the study groups after 1 h.

	Adrenaline (*n* = 21)	Noradrenaline (*n* = 21)	*p*-value
Left ventricle – mean (SD)			
Heart rate (per min)	153.9 (13.3)	152.6 (22.3)	0.83
Velocity time integral (m)	10.0 (0.4)	10.0 (3.2)	0.98
Aortic Outflow Cross Sectional area (AoCSA) (cm^2^)	5.9 (1.2)	6.3 (1.4)	0.44
Output (ml/kg/min)	246.0 (109.2)	292.2 (163.9)	0.33
Fractional shortening (%)	41.8 (21.6)	45.1 (21.1)	0.69
Right ventricle – mean (SD)			
Heart rate (per min)	154.0 (13.3)	154.1 (21.9)	0.99
Velocity time integral (m)	10.9 (2.8)	11.3 (3.5)	0.78
Pulmonary Outflow Cross Sectional area (PoCSA) (cm^2^)	6.2 (1.2)	6.4 (1.4)	0.73
Output (ml/kg/min)	322.6 (158.0)	329.5 (145.3)	0.89
Tricuspid Annular Plane Systolic Excursion (TAPSE) (mm)	8.8 (3.2)	9.8 (2.5)	0.36

The administration of noradrenaline via peripheral venous access was found to be safe with no adverse effects. Tachycardia occurred in 19.0% cases managed by noradrenaline and 28.6% cases managed by adrenaline (*p*-value = 0.77). Incidence of AKI, nosocomial infection, brain hemorrhage, and NEC were similar between the two groups (Table 3). Mechanical ventilation was required in 71.4% and 81.0% of cases in the noradrenaline and adrenaline groups, respectively (*p*-value = 0.34). Duration of hospital stay was similar for the two groups (12 and 15 days, respectively; *p*-value = 0.43).

**Table 3:** Associations of study drugs with outcomes.

	Adrenaline (*n* = 21)	Noradrenaline (*n* = 21)	Odds Ratio (95% Confidence interval)	*p*-value^a^
Primary outcomes				
Resolution of shock within 1 h, *n* (%)	13 (61.9%)	16 (76.2%)	0.71 (0.14–3.64)	0.32
Death, *n* (%)	7 (33.3%)	6 (28.6%)	1.45 (0.39–5.50)	0.74
Secondary outcomes				
Other vasopressor required, *n* (%)	8 (38.1%)	5 (23.8%)	0.62 (0.17–2.25)	0.53
Dose escalation within 2 h required, *n* (%)	6 (28.6%)	9 (42.9%)	0.61 (0.17–2.25)	0.33
Intraventricular hemorrhage, *n* (%)	9 (42.9%)	7 (33.3%)	1.5 (0.43–5.25)	0.75
Nosocomial infection, *n* (%)	8 (38.1%)	4 (19.0%)	2.65 (0.64–10.61)	0.31
Tachycardia, *n* (%)	6 (28.6%)	4 (19.0%)		0.77
Acute kidney injury, *n* (%)	2 (9.5%)	3 (14.3%)	0.63 (0.09–4.23)	1.00
Necrotizing enterocolitis (stage II/III), *n* (%)	2 (9.5%)	1 (4.8%)	2.10 (0.17–25.17)	1.00
Cerebellar hemorrhage, *n* (%)	1 (4.8%)	1 (4.8%)	1.00 (0.06–17.12)	1.00
Periventricular leukomalacia, n	0	0		1.00
Hospital stay (days), median (IQR)	15 (6–33)	12 (5–19)		0.44
NICU stay (days), median (IQR)	10 (5–30)	6 (3–10)		0.04
Mechanical ventilation (days), mean (SD)	1.24 (0.54)	1.29 (0.46)		0.76

^a^ For continuous variables, standard t test, and for categorical variables, chi square test or Fisher's exact test in case of any observed frequency ≤ 5.

## Discussion

Our study showed no difference in resolution of fluid-refractory septic shock within an hour in 76% of neonates treated with noradrenaline as compared to 62% cases treated with adrenaline (*p*-value = 0.53) with no difference in mortality during hospitalisation. Baske et al. reported reversal of such shock in 25% of 20 neonates in the adrenaline group (0.2 to 0.3 to 0.4 μg/kg/min) in their study (6), which is comparable to our study's finding. Similarly, Ramaswamy et al. reported that shock resolved in 48% of 29 children of age 3 months to 12 years with use of adrenaline (0.1 to 0.2 to 0.3 μg/kg/min) (9). In their trial of noradrenaline (0.1–0.3 μg/kg/min) in pediatric septic shock, Tourneux et al. found that hypoperfusion was reversed in 81% of cases within 6 h after starting treatment (10). In a 10-year review study on the use of noradrenaline in pediatric septic shock, Lampin et al. concluded that greater noradrenaline doses than those suggested in the literature may be required to correct hypotension and hypoperfusion (11). Kallimath et al. has described the noradrenaline cohort of this study in detail (12).

The groups in our study were comparable with respect to baseline parameters (Tables 1, 2). Males accounted for 71.4% of our study population of 42 neonates with septic shock. This male predominance, suggesting role of some sex-related element in vulnerability to neonatal sepsis, has been observed by multiple groups (13–15). The mean gestational age and birth weight of our cases were 36.8 weeks and 1.9 kg, with 69% and 80.1% of the neonates being pre-term and small for gestational age, respectively. Prevalence of pre-term birth among indian neonates with sepsis has been reported at 55%–73% (16–18), and neonatal septicemia has been associated with low birth weight (16–21). Seizures (19%), lethargy (17%), feed refusal (14%), and fever were the most common symptoms in our study population, more than half of which were observed to have hypotension (98%), cold peripheries (86%), icterus (81%), pallor (74%), weak pulse (60%), low oxygen saturation (55%), edema (52%), and tachypnea (52%). Seizures (39%), lethargy (27%), reluctance to feed (19%), and fever (6%) were the most common presenting symptoms of neonatal sepsis in one study from India (16). In our study, all cases with septic shock had hypotension and around three quarters of them had cold extremities and a weak pulse.

Tachycardia was the only adverse event observed, occurring in 19% and 29% of cases of the noradrenaline and adrenaline groups, respectively. In neonates with fluid-refractory septic shock treated with adrenaline, Ramaswamy et al. noted a 14% frequency of adverse events, with tachycardia and arterial gangrene occurring in 3% and 10% of cases, respectively (9). We did not observe arrhythmia or hypertension with noradrenaline unlike previous study (11), which were dose related. Although very large doses of noradrenaline were found safe while allowing for a 33% survival rate in adults (22), there is a need for further studies of dose optimization in neonates.

The noradrenaline and adrenaline groups had similar mortality rates (28.6% vs. 33.6%, *p*-value = 0.77) statistically. In the study cohort, the baseline characteristics, clinical and hemodynamic parameters were similar in neonates who survived and those who succumbed. As in previous studies from our unit, we have found pulmonary hypertension in cases of septic shock, we hypothesised that noradrenaline might be beneficial from that perspective where we had cases of late onset sepsis with pulmonary hypertension and high cardiac output (4). Hence, we wanted to study noradrenaline with effects of an vasopressor and a pulmonary vasodilator.

The mortality rates in sepsis in infancy have varied from 18% (10), 27% (16), 37% (23), 45% (11) to 58% (9), depending on the treatment drugs and the age group. These high mortality rates reinforce the need for early sepsis recognition with appropriate management and further research to identify the first line medication and optimal doses.

The study had few limitations. The study sample size could not be reached and it was a underpowered study. Also usage of non-invasive blood pressure recordings with reliability on reduction of blood pressure to less than 10th centile for definition of shock was used. The bolus fluid therapy at 10 ml/kg might be inadequate for defining fluid refractory septic shock.

## Conclusion

In this single centre open labelled study in neonates with fluid-refractory septic shock, noradrenaline was comparable to adrenaline in resolution of shock at one hour of infusion and mortality during hospitalisation. Also, it had similar hemodynamic and adverse reaction profile. The study being underpowered, could not conclude non-inferiority of noradrenaline to adrenaline. Further studies with larger sample sizes to evaluate the efficacy and safety of these two drugs as a first-line agent in neonatal septic shock are required.

## Data Availability

The raw data supporting the conclusions of this article will be made available by the authors, without undue reservation.

## References

[1] Fleischmann C Reichert F Cassini A Horner R Harder T Markwart R Global incidence and mortality of neonatal sepsis: a systematic review and meta-analysis. Arch Dis Child. (2021) 106(8):745–52. (published Online First: 2021/01/24). 10.1136/archdischild-2020-320217 33483376 PMC8311109

[2] Weiss SL Peters MJ Alhazzani W Agus MSD Flori HR Inwald DP Surviving sepsis campaign international guidelines for the management of septic shock and sepsis-associated organ dysfunction in children. Pediatr Crit Care Med. (2020) 21(2):e52–e106. 10.1097/PCC.0000000000002198 32032273

[3] Davis AL Carcillo JA Aneja RK Deymann AJ Lin JC Nguyen TC American college of critical care medicine clinical practice parameters for hemodynamic support of pediatric and neonatal septic shock. Crit Care Med. (2017) 45(6):1061–93. (published Online First: 2017/05/17). 10.1097/CCM.0000000000002425 28509730

[4] Deshpande S Suryawanshi P Holkar S Singh Y Yengkhom R Klimek J Pulmonary hypertension in late onset neonatal sepsis using functional echocardiography: a prospective study. J Ultrasound. (2022) 25(2):233–9. 10.1007/s40477-021-00590-y 33991307 PMC9148354

[5] Banait N Suryawanshi P Malshe N Nagpal R Lalwani S. Cardiac blood flow measurements in stable full term small for gestational age neonates. J Clin Diagn Res. (2013) 7(8):1651–4. 10.7860/JCDR/2013/5671.3302 24086865 PMC3782922

[6] Baske K Saini SS Dutta S Sundaram V. Epinephrine versus dopamine in neonatal septic shock: a double-blind randomized controlled trial. Eur J Pediatr. (2018) 177(9):1335–42. 10.1007/s00431-018-3195-x 29936590

[7] Samanta M Mondal R Ray S Sabui T Hazra A Kundu C Normative blood pressure data for Indian neonates. Indian Pediatr. (2015) 52(8):669–73. 10.1007/s13312-015-0694-y 26388624

[8] Saghaei M. Random allocation software for parallel group randomized trials. BMC Med Res Methodol. (2004) 4:26. (published Online First: 2004/11/13). 10.1186/1471-2288-4-26 15535880 PMC533876

[9] Ramaswamy KN Singhi S Jayashree M Bansal A Nallasamy K. Double-blind randomized clinical trial comparing dopamine and epinephrine in pediatric fluid-refractory hypotensive septic shock*. Pediatr Crit Care Med. (2016) 17(11):e502–e12. 10.1097/pcc.0000000000000954 27673385

[10] Tourneux P Rakza T Abazine A Krim G Storme L. Noradrenaline for management of septic shock refractory to fluid loading and dopamine or dobutamine in full-term newborn infants. Acta Paediatr. (2008) 97(2):177–80. 10.1111/j.1651-2227.2007.00601.x 18177443

[11] Lampin M Rousseaux J Botte A Sadik A Cremer R Leclerc F. Noradrenaline use for septic shock in children: doses, routes of administration and complications. Acta Paediatr. (2012) 101(9):e426–30. 10.1111/j.1651-2227.2012.02725.x 22568565

[12] Kallimath A Garegrat R Patnaik S Singh Y Soni NB Suryawanshi P. Hemodynamic effects of noradrenaline in neonatal septic shock: a prospective cohort study. J Trop Pediatr. (2024) 70(2):fmae001. 10.1093/tropej/fmae001 38324898

[13] Todi S Chatterjee S Sahu S Bhattacharyya M. Epidemiology of severe sepsis in India: an update. Critical Care. (2010) 14(Suppl 1):382. 10.1186/cc8614

[14] Muley VA Ghadage DP Bhore AV. Bacteriological profile of neonatal septicemia in a tertiary care hospital from western India. J Glob Infect Dis. (2015) 7(2):75–7. 10.4103/0974-777X.154444 26069427 PMC4448329

[15] Jyothi P Basavaraj MC Basavaraj PV. Bacteriological profile of neonatal septicemia and antibiotic susceptibility pattern of the isolates. J Nat Sci Biol Med. (2013) 4(2):306–09. 10.4103/0976-9668.116981 24082722 PMC3783770

[16] Kakkar C Galhotra R Saggar K Arora A. Clinico-bacteriological profile of neonatal septicemia in a tertiary care hospital. J Mahatma Gandhi Inst Med Sci. (2015) 20(2). 10.4103/0971-9903.164252

[17] Chugh K Aggarwal BB Kaul VK Arya SC. Bacteriological profile of neonatal septicemia. Indian J Pediatr. (1988) 55(6):961–65. 10.1007/bf02727838 3069720

[18] Anand NK Gupta AK Mohan M Lamba IM Gupta R Srivastava L. Coagulase negative staphylococcal septicemia in newborns. Indian Pediatr. (1991) 28(11):1241–8. (published Online First: 1991/11/01). 1808044

[19] Christo GG Mathai J Nalini B Baliga M Venkatesh A. Neonatal citrobacter sepsis: clinical and epidemiological aspects. Indian J Pediatr. (1990) 57(6):781–84. 10.1007/bf02722277 2131309

[20] Begum S Baki M Kundu G Islam I Kumar M Haque A. Bacteriological profile of neonatal sepsis in a tertiary hospital in Bangladesh. J Bangladesh Coll Physicians Surg. (2012) 30(2):66–70. 10.3329/jbcps.v30i2.11406

[21] Stoll BJ Hansen N Fanaroff AA Wright LL Carlo WA Ehrenkranz RA Late-onset sepsis in very low birth weight neonates: the experience of the NICHD neonatal research network. Pediatrics. (2002) 110(2 Pt 1):285–91. (published Online First: 2002/08/08). 10.1542/peds.110.2.285 12165580

[22] Katsaragakis S Kapralou A Theodorou D Markogiannakis H Larentzakis A Stamou KM Refractory septic shock: efficacy and safety of very high doses of norepinephrine. Methods Find Exp Clin Pharmacol. (2006) 28(5):307–13. (published Online First: 2006/07/18). 10.1358/mf.2006.28.5.990203 16845448

[23] Tallur SS Kasturi AV Nadgir SD Krishna BVS. Clinico-bacteriological study of neonatal septicemia in hubli. Indian J Pediatr. (2000) 67(3):169–74. 10.1007/bf02723654 10838717

